# From the 1990s climate change has decreased cool season catchment precipitation reducing river heights in Australia’s southern Murray-Darling Basin

**DOI:** 10.1038/s41598-021-95531-4

**Published:** 2021-08-09

**Authors:** Milton S. Speer, L. M. Leslie, S. MacNamara, J. Hartigan

**Affiliations:** School of Mathematical and Physical Sciences, University of Technology, Broadway, PO Box 123, Sydney, NSW 2007 Australia

**Keywords:** Climate-change ecology, Atmospheric dynamics, Climate change

## Abstract

The Murray-Darling Basin (MDB) is Australia’s major agricultural region. The southern MDB receives most of its annual catchment runoff during the cool season (April–September). Focusing on the Murrumbidgee River measurements at Wagga Wagga and further downstream at Hay, cool season river heights are available year to year. The 27-year period April–September Hay and Wagga Wagga river heights exhibit decreases between 1965 and 1991 and 1992–2018 not matched by declining April-September catchment rainfall. However, permutation tests of means and variances of late autumn (April–May) dam catchment precipitation and net inflows, produced p-values indicating a highly significant decline since the early 1990s. Consequently, dry catchments in late autumn, even with average cool season rainfall, have reduced dam inflows and decreased river heights downstream from Wagga Wagga, before water extraction for irrigation. It is concluded that lower April–September mean river heights at Wagga Wagga and decreased river height variability at Hay, since the mid-1990s, are due to combined lower April–May catchment precipitation and increased mean temperatures. Machine learning attribute detection revealed the southern MDB drivers as the southern annular mode (SAM), inter-decadal Pacific oscillation (IPO), Indian Ocean dipole (IOD) and global sea-surface temperature (GlobalSST). Continued catchment drying and warming will drastically reduce future water availability.

## Introduction

**Figure 1:**
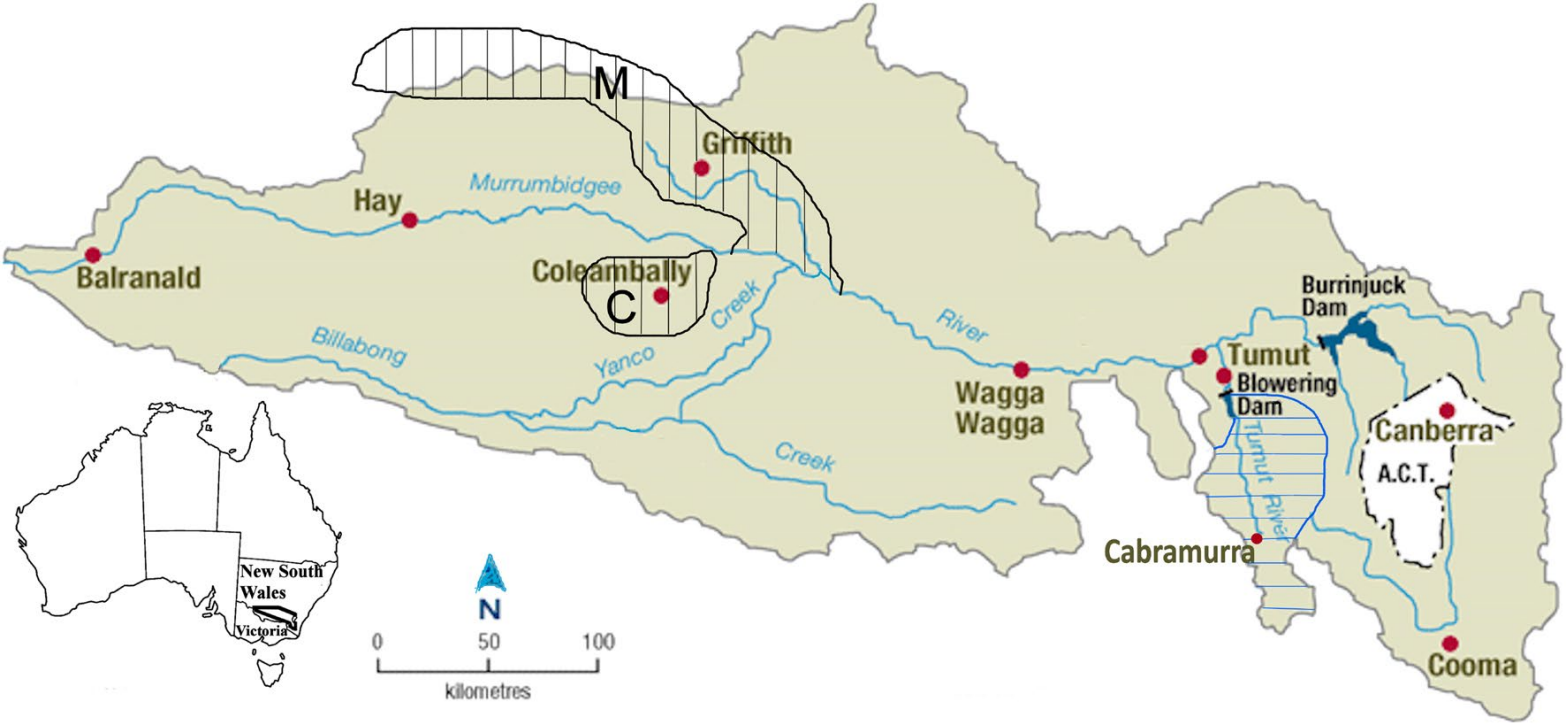
The Murrumbidgee River and catchment area. *M* Murrumbidgee irrigation area (hatched black), *C* Colleambally irrigation area (hatched black). Snowy mountains hydroelectric scheme (hatched blue). The inset identifies the catchment location in southeast Australia. Catchment area stations are Blowering Dam, Burrinjuck Dam and Tumut (Created in Corel Paintshop Pro, Version 23.1.0.27).

The Murray-Darling River basin (MDB), located in southeastern Australia, is Australia’s most important agricultural area, producing almost 40% of the national food supply^1^. In comparison, the agricultural area of the central valley of California provides approximately 20% of the total US food production^2^. The extended drought conditions since the mid-1990s in the MDB, comprising the Millennium Drought (1997–2009)^3,4^; and the 2017–2019 drought, have resulted in rivers running dry and low water storages, affecting communities, businesses, animals, and the environment. The southern MDB, in which the Murrumbidgee River catchment is located (Fig. 1), occupies a large geographical area of southeast Australia where the main growing season is in the cooler half of the year (April-September)^5^. Most of the southern MDB in Victoria close to the Murrumbidgee River catchment (Fig. 1) continues to suffer hydrological drought despite the easing of the meteorological Millennium Drought in 2010^6^.

## Murrumbidgee river heights

**Figure 2:**
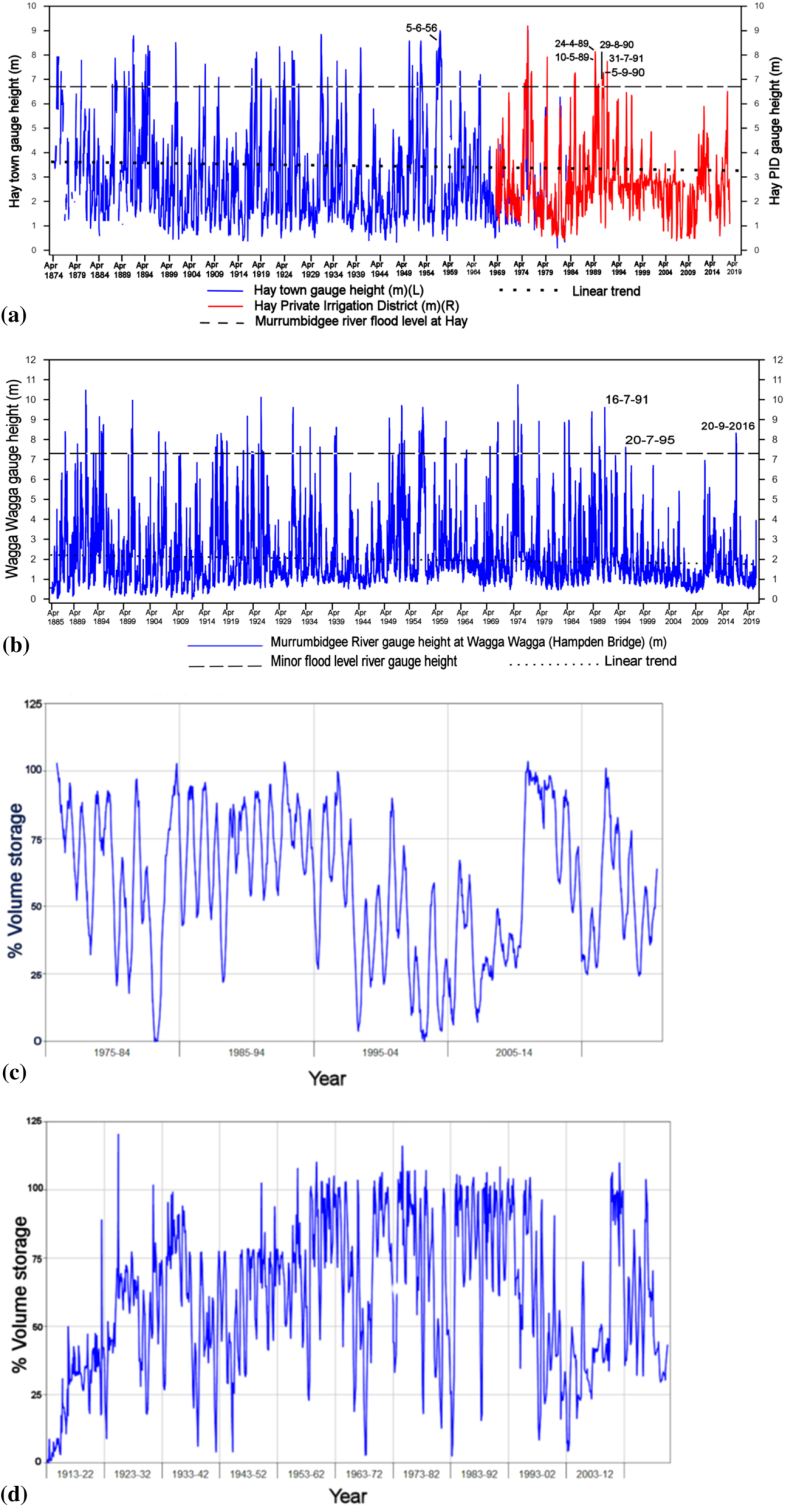
Murrumbidgee river heights at Hay and Wagga Wagga; annual inflows at Blowering Dam and Burrinjuck Dam. (**a**) Murrumbidgee river heights (m) at the Hay town gauge and Hay PID for (April–September) 1874 to 2019. There are concurrent readings at the two locations from 1968 to 1982. The first April height every 5 years is indicated. The river flood level is marked (dashed black line) at 6.7 m. Of the six months April-September the most recent maximum weekly river height value for each individual month exceeding the flood level of 6.7 m, are designated. Note that since 1991 there have no April–September flood height exceedance peaks and the most recent June flood height exceedance was 5 June 1956, (**b**) Murrumbidgee river heights at Wagga Wagga (April to September) 1885–2019. The minor flood level is marked (dashed black line) at 7.3 m. Note that since 1991 there has been only one minor flood level reached in 1995 and one major in 2016, (**c**) annual volume storage (%) for Blowering Dam indicating precipitation inflows from 1975 to 2019. Note that there is a rapid increase followed by a long decline as a result of the 2010–2012 La Niña and in 2016 as a result of a strong IOD influence, (**d**) annual volume storage (%) for Burrinjuck Dam indicating precipitation inflows from 1913 to 2019. Note the similar rapid increase in 2010 followed by a long decline from 2012 and in 2016, as in (**c**).

To ensure the supply of precipitation-driven inflows, it is crucial to receive average or close to average rainfall during the global warming period. In and around the Murrumbidgee catchment Burrinjuck Dam and Blowering Dam receive 57%, Tumut 56%, Wagga Wagga 52% and Hay 52% of their annual average rainfall in this period. Notably, stream flows have been declining across the MDB since the 1990s (Figs.2a,b).

## Drying trends in the Murray-Darling Basin

Aside from the 2017–2019 drought, the MDB previously experienced major multi-year droughts in 1997–2009 (Millennium Drought), 1938–1946 (World War II Drought) and 1895–1903 (Federation Drought). There are downward trends in rainfall since the 1990s in southern parts of eastern Australia, and in the higher rainfall areas of the MDB catchments that generally are located in the highlands of the Great Dividing Range^5^. These trends are concentrated in the cooler half of the year, from April to September^3^. A major influence on this drying trend has been the strengthening and extension of the subtropical high pressure ridge during winter, which shifts rain-bearing weather systems southward^5,6^. Specifically, the lack of negative-phase Indian Ocean Dipole (IOD) events has been identified as a contributing factor to the drying and resulting droughts in southeast Australia since the 1990s^7^, where the influence of the IOD is greatest in June–October^8^**.** Other important climate drivers of cool season precipitation are the atmospheric-based southern annular mode (SAM), which in its positive phase is also a major influence on the drying trend, by its association with rain-bearing systems shifting southward, away from southern Australia^3,4^, and the basin-wide Pacific Ocean phenomenon, the inter-decadal Pacific oscillation (IPO). These and other possible climate drivers are assessed in the Results Section, using Machine Learning (ML) techniques to attribute identification.

## Effect of regulation on river heights

Prior to the 1990s, the main regulation of the Murrumbidgee River upstream of Hay occurred over two periods during which large storages were built. These are the Burrinjuck Dam in 1910–1927, which started filling during the construction phase and Blowering Dam 1956–1961 (Figs. 1, 2c,d). Before the Burrinjuck Dam was built, annual river flows at Hay followed a pattern of variability and quantity similar to annual river flows measured at Wagga Wagga (Fig. 1). After the Burrinjuck Dam was built (1928), the water flows diverged in quantity although their annual variability patterns coincided^9^. After the Blowering Dam construction was completed in the 1960s, the relationship between river flows at Wagga Wagga and Hay were affected. There were similar patterns of variability but there was a substantial difference in the quantity of water reaching Hay from Wagga Wagga, compared with that before river regulation^9,10^. It was concluded that there was a 56% reduction in the lower Murrumbidgee River at Hay^11^, which occurred mostly after 1957 when there was a change point between low regulated flow and highly regulated flow. Even after the 1957 change point, river flows were seasonal, driven by reliable winter and spring rainfall, together with headwater ice and snow melt, and much of the flow reached the floodplain below Hay^11^. However, there has been a sharp drop to zero in JJAS Murrumbidgee River flooding events at Hay since 1991 (Fig. 2a), with just one minor flood in 1995 at Wagga Wagga and one major flood in 2016 (Fig. 2b), in addition to much reduced dam inflows since the mid-1990s (Fig. 2c,d).

In Fig. 2a there is a data overlap of approximately 13 years at Hay town gauge and Hay Private Irrigation District (PID) gauge for which the coefficient of determination (R^2^ = 0.9235), obtained from linear regression of the weekly river height values (N = 668), explains 92% of the variance in the river heights between the two locations. The data quality is high as it takes into account missing values, outliers, continuity between the two gauge locations, and measurement practice^12^. The flooding level of 6.7 m at Hay is defined as the level above which water starts to spill over the riverbanks at Hay^12^ and the minor flood level at the river gauge at Wagga Wagga is 7.3 m.

It is hypothesized here that from the early 1990s the main influence on decreased downstream river water availability is due to the reduction in dam net inflows from decreased precipitation. It is well-known that rainfall in southeast Australia, where the Murrumbidgee River catchment is located, usually is highest in winter and has a minimum through summer into early autumn^5^. Hence the so-called ‘winter cropping season’ starts with planting in autumn and harvesting in spring–summer.

## Monthly-seasonal maximum river flood heights at Hay 1874–2019

Most river height exceedances above 6.7 m at Hay in JJAS occur in late winter (July, August) and September (Table 1) after sufficient catchment wetting. Spring (SON) floods are the most common (Table 2) as river and dam inflows are a result of both rainfall and snowmelt. However, since 1991 there has been just one April to September flood at both Hay and Wagga Wagga resulting from Burrinjuck Dam overspill which was in September 2016 (Table 2). Extreme SON precipitation totals are associated with La Niña over eastern Australia^13^. In terms of river flood events at Hay and Wagga Wagga, and net inflows at the catchment dams of Burrinjuck and Blowering in southeast Australia, this relationship broke down, as shown by the presence only of neutral phases of ENSO in SON after the 1970s (Table 2) and in agreement with eastern Australian rainfall in general^13^. However, there was a strong relationship with ENSO for the 2010 flood in summer (DJF).

**Table 1:** Table showing wet season (JJAS) monthly maximum weekly Murrumbidgee River heights at Hay (southern MDB) that exceed/equal flood level (6.7 m), and seasonal count, in the 125 years 1874–2018. Note the absence of flood height exceedance values since 1991.

Year	Jun	Jul	Aug	Sept	JJAS count
1874		7.92	7.92	7.92	3
1875	7.25	7.32			2
1876				8.0	1
1887		7.86		7.22	2
1889		7.32		7.31	2
1890					
1891		8.78	7.62	6.9	2
1893		6.86	8.3		2
1894		6.71	8.38	8.3	3
1905			6.74		1
1906				7.62	1
1909				7.08	1
1916				7.43	1
1917			7.85	8.1	2
1918			6.89	8.1	2
1922			8.33		
1923		6.94	7.69	6.7	3
1925	8.15	7.77		7.77	3
1926			6.81		1
1931	8.33	8.83	6.7		3
1932				7.08	1
1934				7.74	1
1936			7.39		1
1939				8.2	1
1949			7.56		1
1950			7.55		1
1951				6.85	1
1952	8.22	8.56	7.62		3
1955				8.15	1
1956	8.31	8.99	8.78	7.84	4
1960			7.35	6.82	2
1964			6.93	7.19	2
1974		7.24	7.35	8.99	3
1984			7.2	7.26	2
1989		6.72	6.8		2
1990		6.96	7	7.27	3
1991		7.73			1

**Table 2:** Table showing spring (SON) weekly maximum Murrumbidgee river heights at Hay (southern MDB) that exceed flood level (6.7 m), and seasonal count, in the 125 years 1874–2018.

Year	Sep.	Oct.	Nov.	SON total count	ENSO strength & sign
1874	7.92	7.77		2	N/A
1877			8.17	1	Mod. −
1878		8.99	7.92	2	Mod-strong +
1879	8.0			1	Very strong +
1886	6.95			1	Mod +
1887	7.22			1	Very strong +
1888		7.65		1	Mod. −
1889	7.31			1	Mod. +
1890	6.7			1	Strong +
1891	6.9	7.16	7.01	3	Weak −
1893		7.25		1	Mod-strong +
1894	8.3			1	Very strong +
1905		8.08		1	Mod. −
1906	7.62	8.08		2	Mod. +
1909	7.08			1	Weak −
1916	7.43	8.2		2	Mod. +
1917	8.11	8.31	8.31	3	Very strong +
1918	7.01			1	Weak −
1923	6.7	6.93		2	Mod. −
1925	7.77			1	Mod −
1932	7.08			1	Weak −
1934	7.74		8.29	2	Neutral
1939	8.29			1	Weak −
1950			8.31	1	Mod-strong +
1951	6.85	7.54		2	Neutral
1952		7.8	7.8	2	Neutral
1955	8.15		7.13	2	Mod. +
1956	7.84	7.96	8.23	3	Weak +
1959			7.07	1	Neutral
1960	6.82	8.41		2	Weak +
1964	7.19	8	7.35	3	Weak +
1970		8.56		1	Weak +
1974	8.99	8.63	8.64	3	Weak +
1975			8.62	1	Mod-strong +
1976			7.71	1	Neutral
1978		7.6		1	Neutral
1984	7.26	6.76		2	Neutral
1990	7.27			1	Neutral
2016		8.45		1	Neutral

Also included is the strength of the ENSO signal as determined by the Australian Bureau of Meteorology (http://www.bom.gov.au/climate/enso/lnlist/index.shtml). Note the neutral ENSO association with flood heights since the mid-1970s and only one event since 1990.

In autumn (MAM) there are very few flood events (Table 3) because the catchment typically dries out sufficiently through summer to reduce river flow.

**Table 3:** Table showing autumn (MAM) monthly maximum weekly Murrumbidgee River heights at Hay (southern MDB) that exceed flood level (6.7 m), and seasonal count in the 126 years 1874–2019.

Year	Mar	Apr	May	MAM count
1888		6.8		1
1894		7.32	8.02	2
1950		8.5	7.04	2
1956		6.89	8.23	2
1974			6.85	1
1989		8.41	7.93	2
2012	8.6			1

Note the last April–May flood exceedance height in 1989 and one in March.

## Precipitation time series in the Murrumbidgee river catchment area

**Figure 3:**
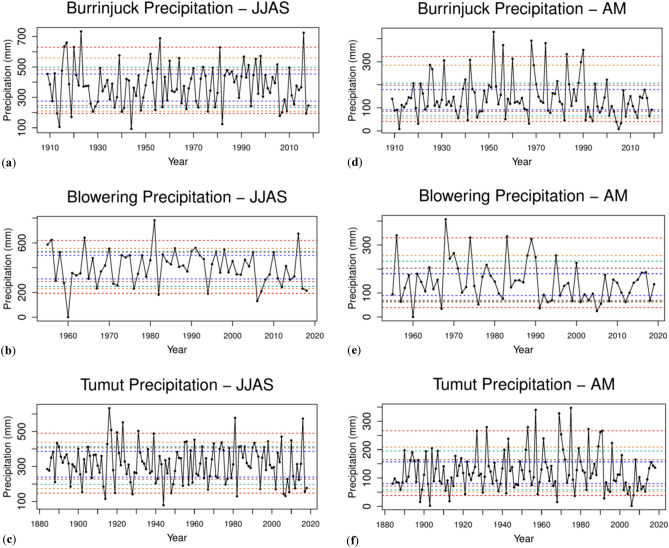
Precipitation time series in the Murrumbidgee River catchment area. JJAS precipitation at, (**a)** Burrinjuck Dam from 1910 to 2019, (**b)** Blowering Dam from 1955 to 2019 and (**c)** Tumut from 1883 to 2019; April–May precipitation at, (**d)** Burrinjuck Dam from 1910 to 2019, (**e)** Blowering Dam from 1955 to 2019 and, (**f)** Tumut from 1883 to 2019. Dashed lines indicate percentiles 5th and 95th (red); 10th and 90th (orange); 15th and 85th (light blue); 20th and 80th (brown); and 25th and 75th (dark blue). Note the decreasing April–May precipitation at the three stations since the early 1990s.

Representing major dam storages, Burrinjuck Dam and Blowering Dam Figs. 3(**a**,**b**) each receives a mean JJAS precipitation of about 300 mm, whereas Tumut receives approximately 350 mm (Fig. 3c). There are fewer percentiles near the extremes from the 1990s, with no years above the 95th percentile apart from 2016 and a reduction in years below the 5th percentile. Statistical significance is discussed in the next section. Similarly, for April–May precipitation there are no years above the 90th percentile from the late 1990s for the three locations (Fig. 3d–f).

All three locations, particularly Burrinjuck Dam and Blowering Dam, are exposed to topographically influenced precipitation from mid-latitude wet season (JJAS) cold frontal weather systems.

Monthly to seasonal precipitation typically exhibits large ranges due to its episodic nature, whereas seasonal means show little change. Therefore, an important part of this study is to analyse trends in both the means and variances of catchment rainfall and inflows, in addition to JJAS river heights at both Hay and Wagga Wagga. The focus is on the period after the 1960s, since there has been no new regulatory infrastructure upstream of Hay. The main focus is from the early to mid-1990s corresponding to the accelerated global warming signal^14^.

Considering changes in regulation of the Murrumbidgee River, box and whisker plots and permutation testing are used to analyse changes in both JJAS and April–May inflows at Burrinjuck/Blowering Dams, and precipitation at key rainfall catchment locations including Burrinjuck Dam, Blowering Dam and Tumut, in addition to river heights at both Hay and Wagga Wagga. The 27-year period chosen for the time series analysis is justified in the Methods Section. For the April–May and JJAS Burrinjuck Dam, Blowering Dam and Tumut combined precipitation, ML techniques are used to identify possible key climate drivers of Murrumbidgee River catchment precipitation. Wavelets then are used to provide some further understanding of possible climate drivers affecting precipitation and net inflows at those locations, and how their influence has changed over time.

## Results

**Figure 4:**
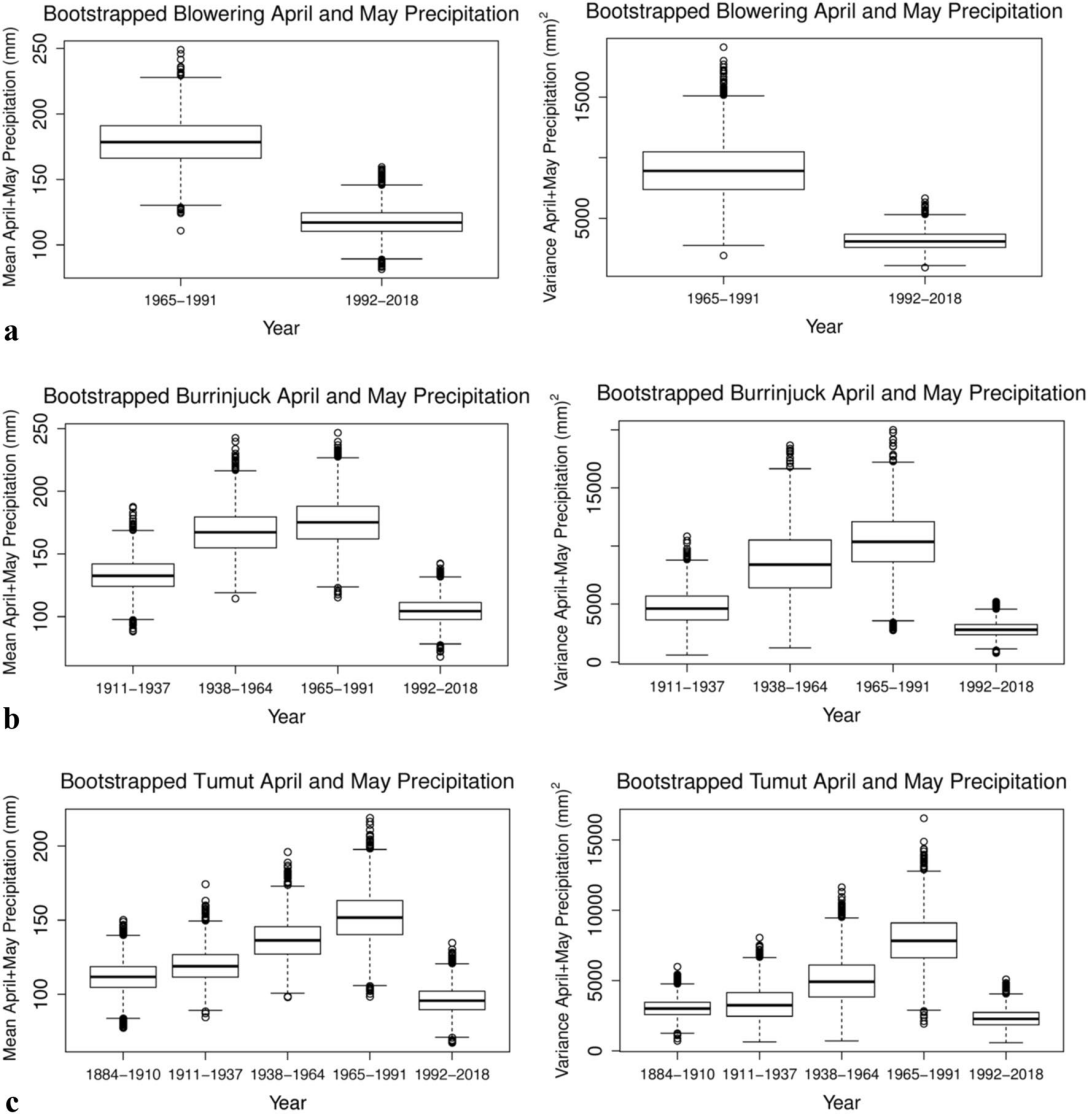
Boxwhisker plots representing mean and variance of April–May Murrumbidgee catchment precipitation. Bootstrapped 27-year intervals of mean (left panel) and variance (right panel) of April–May precipitation for, **(a)** Blowering Dam, **(b)** Burrinjuck Dam, **(c)** Tumut.

For each of the Burrinjuck Dam, Blowering Dam and Tumut time series there has been no statistically significant trend after bootstrapping and box plotting the precipitation into 27-year intervals, for 1965–1991 and 1992–2018. While their JJAS means appear to have decreased slightly (not shown), no locations have observed statistically significant changes (Table 4). For the Tumut JJAS data, precipitation was significantly more variable prior to 1965 (p-values = 0.045 and 0.0984; Table 4). However, for all three locations, their mean late autumn (April–May) precipitation shows a decline in both the mean and variance in the 27-year boxplots between 1965 and 1991 and 1992–2018 (Fig. 4a–c). Statistically, there is a highly significant decrease in both their means (p-values < 0.01; Table 4), and their variances (p-values ~ 0.05; Table 4). It is noteworthy that while neither the mean nor variance of Tumut April–May precipitation between 1884 and 1910 and 1992–2018 is significant, their variances are similarly very low (Fig. 4c-right panel), owing to the very low rainfall that occurred during the Federation Drought (1895–1903).

**Table 4:** P-values from permutation testing differences in interval means and variances for, April–May and JJAS precipitation for Blowering Dam, Burrinjuck Dam, and Tumut; inflows into Blowering Dam and Burrinjuck Dam; Murrumbidgee River heights at Blowering Dam, Burrinjuck Dam, Wagga Wagga and Hay.

Catchment location	Precipitation				Net Inflows (% Volume)				Murrumbidgee River level			
	Apr–May		JJAS		Apr–May		JJAS		Apr–May		JJAS	
	Mean	Var	Mean	Var	Mean	Var	Mean	Var	Mean	Var	Mean	Var
**Blowering Dam**												
1965–1991 vs 1992–2018	0.007*	0.012*	0.202	0.978	–	–	–	–	–	–	–	–
1976–1997 vs 1998–2019	–	–	–	–	0.0348*	0.301	0.0018*	0.0714*	0.0346*	0.426	0.0024*	0.087*
**Burrinjuck Dam**												
1911–1937 vs 1992–2018	0.102	0.237	0.918	0.422	–	–	–	–	–	–	–	–
1938–1964 vs 1965–1991	0.772	0.644	0.846	0.424	0.145	0.093*	0.058*	0.352	0.357	0.268	*0.094*	0.605
1938–1964 vs 1992–2018	0.0034*	0.0486*	0.905	0.818	0.566	0.391	0.104	0.0708*	0.476	0.544	0.0706*	0.051*
1965–1991 vs 1992–2018	0.0024*	0.002*	0.761	0.635	0.0676*	0.517	*0.003*	0.328	0.148	0.606	0.0026*	0.115
**Tumut**												
1884–1910 vs 1938–1964	0.149	0.289	0.463	0.045*	–	–	–	–	–	–	–	–
1938–1964 vs 1965–1991	0.528	0.278	0.308	0.984	–	–	–	–	–	–	–	–
1884–1910 vs 1992–2018	0.285	0.46	0.353	0.008*	–	–	–	–	–	–	–	–
1965–1991 vs 1992–2018	0.0062*	0.0026*	0.227	0.505	–	–	–	–	–	–	–	–
**Wagga Wagga**												
1911–1937 vs 1938–1964	–	–	–	–	–	–	–	–	–	–	0.399	0.55
1911–1937 vs 1992–2018	–	–	–	–	–	–	–	–	–	–	0.0002*	0.013*
1938–1964 vs 1965–1991	–	–	–	–	–	–	–	–	–	–	0.374	0.275
1965–1991 vs 1992–2018	–	–	–	–	–	–	–	–	–	–	0.0044*	0.095*
**Hay**												
1911–1937 vs 1965–1991	–	–	–	–	–	–	–	–	–	–	0.19	0.683
1911–1937 vs 1992–2018	–	–	–	–	–	–	–	–	–	–	0.0068*	0.001*
1938–1964 vs 1965–1991	–	–	–	–	–	–	–	–	–	–	0.309	0.332
1965–1991 vs 1992–2018	–	–	–	–	–	–	–	–	–	–	0.236	0.005*

Significant values (p < 0.10) are italicized with an asterisk. Note that the p-value for each variance test is calculated after one sample has had bias correction in the mean. Note that the p-value for each variance test is calculated after one sample has had bias correction in the mean. Key points to note are highly significant April–May precipitation decreases from 1965 to 1991 to 1992–2018, highly significant recent JJAS net inflows into Blowering and Burrinjuck Dams, and some significant recent decreases in mean or variance of JJAS river heights at Wagga Wagga and Hay.

There is an apparent decrease in the mean and variance of JJAS Murrumbidgee River heights at Hay between the bootstrapped intervals 1938–1964 to 1965–1991 highlighted by their low p-values (Table 4; figure not shown), which corresponded to a change from low to highly regulated river flow^11^. However, the apparent mean and variance decrease is not statistically significant, whereas the variance between the 27-year bootstrapped intervals 1965–1991 to 1992–2018 is highly significant (p-value ~ 0.005; Table 4). The mean river height in this period for Hay (p = 0.236; Table 4) is not statistically significant most likely due to snow melt starting to reach Hay in September. Both Hay and Wagga Wagga river heights are significantly lower in both mean and variance from 1911 to 1937 to 1992–2018 owing to a high rainfall period 1911–1937 compared to the period 1992–2018 (p-values = 0.0068, 0.001, respectively for Hay; 0.0002, 0.013, respectively for Wagga Wagga). For Wagga Wagga the decrease in both the mean and variance from 1965 to 1991 to 1992–2018 is highly significant (p-values ~ 0.0068 and 0.001, respectively; Table 4). Importantly, the significant mean decrease at Wagga Wagga suggests that the cause is climate related with river water used for irrigation not a major contributor since almost all irrigation occurs downstream between Wagga Wagga and Hay. There is no significant change in October–March precipitation mean or variance from 1965 to 1991 to 1992–2018 for the three catchment rainfall locations (p-values not shown).

**Figure 5:**
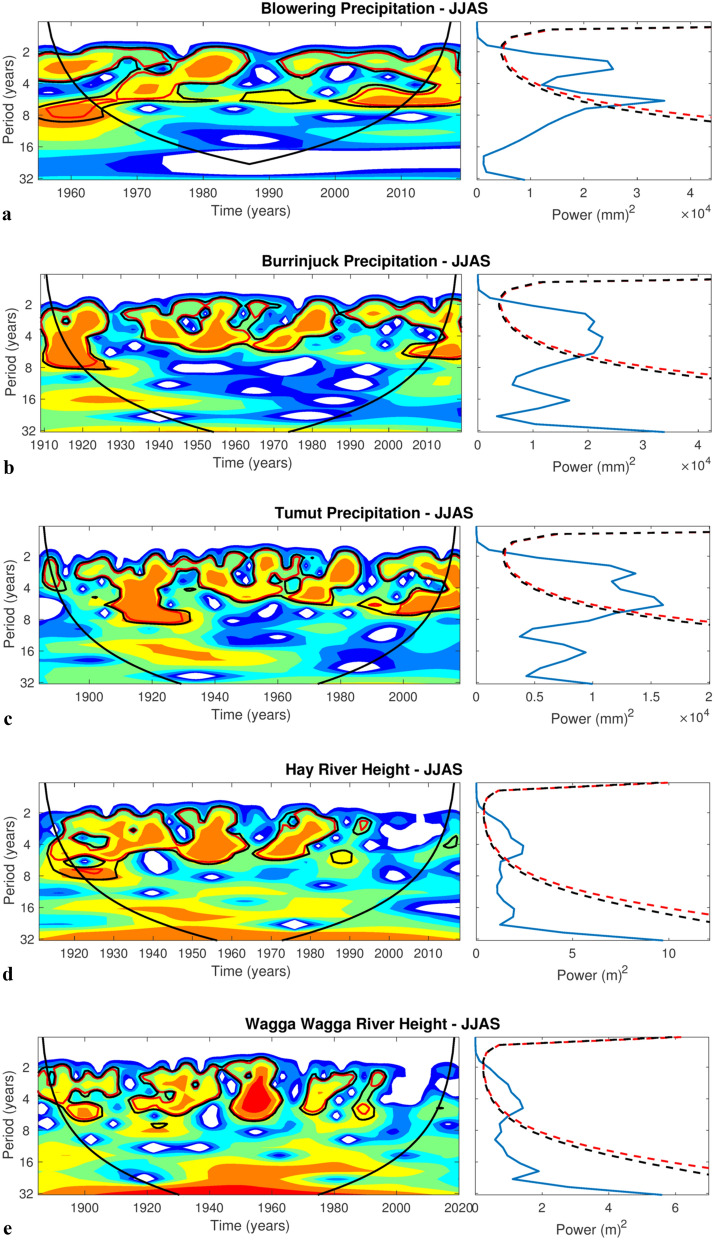
Wavelets of JJAS precipitation and river height. JJAS wavelets and global power spectra of precipitation for, (**a)** Burrinjuck Dam, (**b)** Blowering Dam, (**c)** Tumut; and (**d)** JJAS mean weekly Murrumbidgee river heights at Hay. Power significance is indicated by areas within thick black or red contours the 5% or 10% confidence levels, respectively.

The mean JJAS precipitation wavelets for Burrinjuck Dam, Blowering Dam, Tumut and the mean JJAS weekly Murrumbidgee River heights at Hay (Fig. 5a–d), respectively, exhibit a significant ENSO-like periodicity of approximately 2–7 years, which mostly weakens in the three precipitation locations from the 1990s, and, in the case of the river heights at Hay, disappears after the 1990s. A possible reason for the weakening ENSO-like periodicity is that the nonlinear ENSO amplitude has weakened (less strong El Niños) in response to global warming^15,16^.

There is a clear periodicity of 2–7 years, suggestive of the ENSO time scale, at the three catchment precipitation locations (Fig. 5a–c). However, the ENSO signal disappears from the late 1990s for the downstream river heights at Wagga Wagga and Hay (Fig. 5d,e). This period corresponds to a lack of major precipitation-driven net inflows except for 2010–2012 and 2016.

Apart from ENSO there are many potential climate drivers that could influence precipitation in the Murrumbidgee catchment, and therefore river heights along the Murrumbidgee River. Potential attributes considered in this study include the IOD, global sea surface temperature anomalies (GlobalSSTA), global temperature anomalies (GlobalT), Niño3.4, the IPO, the SAM, the Southern Oscillation Index (SOI), and Tasman Sea sea surface temperature anomalies (TSSST). Additionally, two-way interaction terms between these predictors were considered as potential attributes, obtained through multiplication of two variables with each other (e.g., SAM*IPO). For April–May precipitation, the important attributes were assessed as GlobalT, SAM, SOI, GlobalT*SAM and GlobalT*IPO. For JJAS precipitation, the key attributes were IOD, SAM, SOI, IPO and GlobalT*GlobalSSTA.

**Figure 6:**
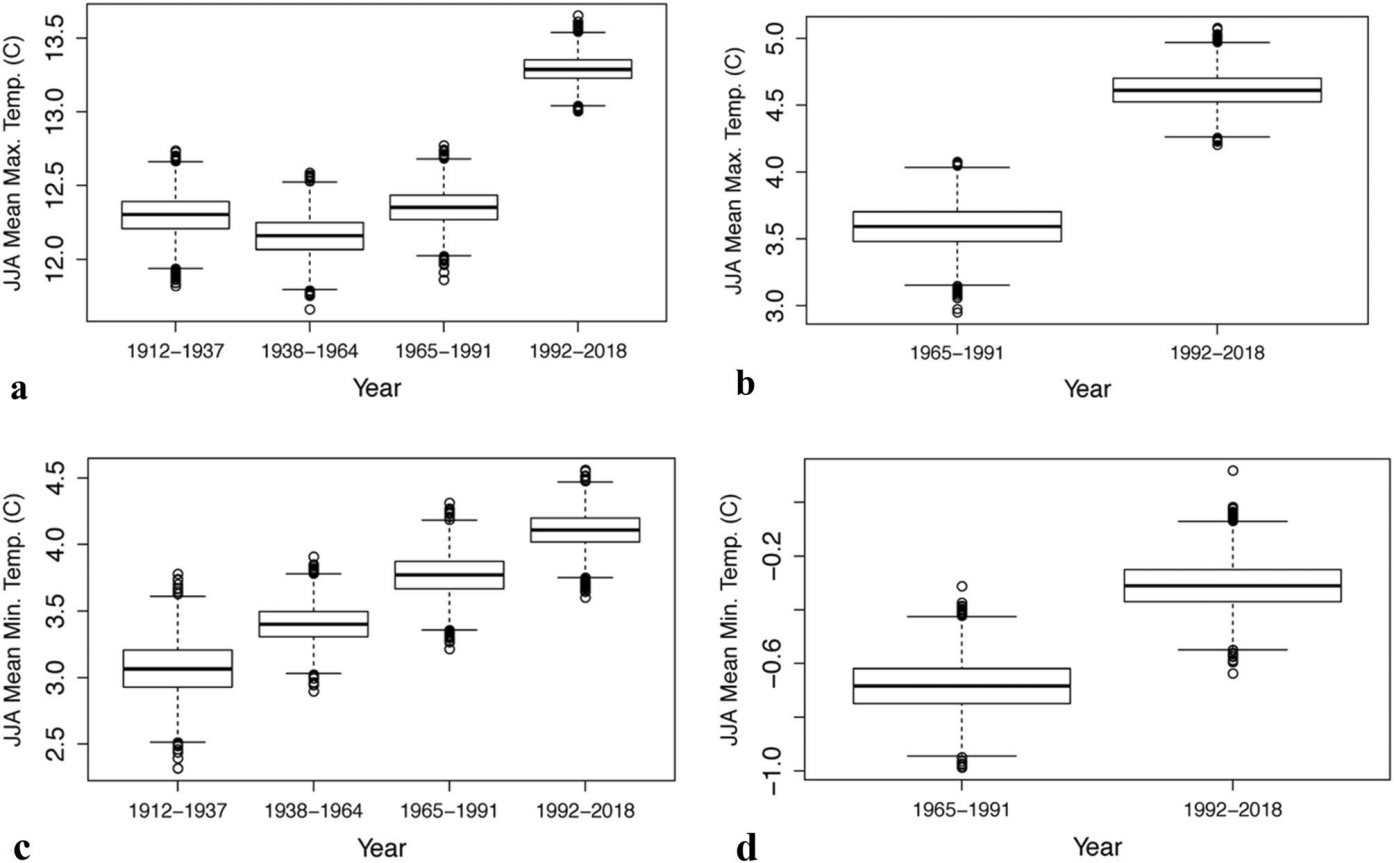
Box plots of mean temperature in the Murrumbidgee River catchment.** (a)** Bootstrapped 27-year interval Burrinjuck JJA mean TMax (C), (**b)** bootstrapped 27-year interval Cabramurra JJA mean TMax (C), (**c)** bootstrapped 27-year interval Burrinjuck Dam JJA mean TMin (C), and (**d)** bootstrapped 27-year interval Cabramurra JJA mean TMin (C). Note the highly significant increase in JJAS mean maximum temperatures at both Burrinjuck Dam and Cabramurra from 1965 to 1991 to 1992–2018 and the increasing trend in mean minimum temperatures.

There is a clear increasing trend in JJAS mean TMax and TMin at both Burrinjuck Dam and Cabramurra (located close to Blowering Dam), revealed by their 27-year interval boxplots (Fig. 6a–d), and consistent with the known global warming signal since 1950^17^ which has accelerated in the last 50 years^18,19^ and particularly since the mid-1990s in Australia^13^. This is confirmed by the very high levels of significance (all p-values are < 0.05) for the difference in TMax and TMin means between the intervals 1965–1991 to 1992–2018 (Table 5).

**Table 5:** P-values from permutation testing differences in JJAS mean maximum. (TMax) and mean minimum (TMin) temperature (C) relative to 27-year bootstrapped intervals for Burrinjuck Dam and Cabramurra (nearby surrogate for Blowering Dam).

27-year bootstrapped interval	Statistic	JJAS Burrinjuck Dam P-values		JJAS Cabramurra P-values	
		TMax	TMin	TMax	TMin
1965–1991 vs 1992–2018	Mean	0*	0.0452*	0*	0.002*
	Variance	0.849	0.926	0.613	0.647

Significant values (p < 0.05) are italicized with an asterisk.

Cabramurra (altitude 1482 m) shows a highly significant increase in TMin between the two bootstrapped intervals in contrast to a non-significant increase at Burrinjuck Dam (altitude 390 m). With such a large difference in altitude between the two locations, it is possible that other factors may be involved, including local differences in meteorological variables such as wind speed/wind direction and/or cloud cover.

## Discussion

The JJAS river heights at Hay (Fig. 2a) have clearly reduced variability over the 27-year period 1965–1991 compared with 1992–2018 (p-value = 0.005, Table 4). Despite the apparent decrease in the mean and variance of JJAS Murrumbidgee River heights at Hay from the 1960s, as shown by the low p-values (0.309, 0.332), respectively, for the 27-year intervals 1938–1964 to 1965–1991 (Table 4), the decrease is not statistically significant. However, the decrease is consistent with the suggestion that a change point occurred from the late 1950s between unregulated to regulated flow at Hay^11^.

While the mean JJAS precipitation of the three catchment locations of Burrinjuck Dam, Blowering Dam and Tumut indicate a slight decrease in percentile extremes from the 1990s, with 2016 (due to September) only above the 95th percentile (Fig. 3), they exhibit no significant change in mean or variance based on the bootstrapped intervals JJAS 1964–1991 to 1992–2018. Consequently, the question arises of why the significant decrease in mean and variance of the JJAS Murrumbidgee River height at Wagga Wagga and in variance at Hay does not match a similar significant decrease in mean or variance of JJAS catchment rainfall. A rainfall decline in recent decades was found to be most pronounced in late autumn^20,21^ and that without sufficient autumn rainfall to moisten catchments in southern Australia, follow-up rainfall in winter cannot be efficiently converted to run-off and catchment inflows^22^. There have been statistically significant decreases in April–May mean precipitation at the catchment locations of Blowering Dam, Burrinjuck Dam and Tumut from 1964 to 1991 to 1992–2018 and also for the mean inflows to the two Dams (Table 4). Furthermore, as a result of the Millennium Drought (1997–2009), modelling experiments indicate that, starting from very dry conditions, the run-off response to rainfall only will return to the normal pre-drought conditions after about 10–20 years of average rainfall^23^. Therefore, the significant decrease in variance of Murrumbidgee River heights at Hay and in mean and variance at Wagga Wagga, is most likely due to the April–May reduced dam inflows and precipitation, and from average JJAS catchment precipitation since 1991. Any role played by water extraction for irrigation between Wagga Wagga and Hay, where irrigation is concentrated, is likely to be small owing to the highly significant mean river height reduction at Wagga Wagga which is upstream from Hay. However, irrigation, and other water usage, is sourced from the dams, so there is a long-term impact of irrigation over the months preceding JJAS on flows at Wagga Wagga, due to the reduction in water stored in the upstream dams. The dams integrate the water extracted for irrigation and all other usage since the last spill event, and therefore the extractions over an extended period can have an impact on when the dam will fill, and hence on the flows downstream, including Wagga Wagga. The minimum water level in the dams, which typically occurs near the end of Autumn, is due to the reduction in inflows (impacted by climate change), and extractions from the dam. Coupled with the tendency for a slower fill rate due to reduced inflows, this results in fewer spill events. As a consequence, there is a change in the distribution between spill events and irrigation releases, changing the frequency distribution of flows. This will be particularly the case for the JJAS period, as a delayed dam filling will have a major impact in dam levels in that period. Before the 1990s the river at Wagga Wagga and Hay reached flood level height or close to flood level height regularly in JJAS from precipitation-driven inflows regardless of the amount of water that was extracted (see Fig. 2a,b). Since the mid-1990s less water reaching Wagga Wagga has significantly reduced the river height owing to significantly decreased April–May and JJAS precipitation-driven inflows at the upstream catchment dams of Burrinjuck and Blowering Dams and significantly decreased mean precipitation at Tumut, which also represents the catchment area of Blowering Dam (Fig. 2c,d; Table 4). Moreover, there has been no overallocation or hoarding of water found in the southern MDB^24^. In a different southern MDB catchment study of the Millennium Drought 1997–2008, factors for a disproportionate reduction in rainfall run-off were reduced mean annual rainfall, less interannual variability of rainfall, changed seasonality of rainfall and lastly increased potential evaporation^25^. However, the last two factors mentioned have since become well established in the last decade with reference to the work in this study. It was suggested that a rainfall reduction alone does not explain the observed inflow reduction trend^26^. Even after a major rain event, the soils are so dry that they absorb more water than before the rain event, and less reaches the dams and rivers than on a wet catchment. In the last three decades it is unknown what the effect on run-off into dams and the Murrumbidgee river has been in JJAS from major rain events because, apart from August–September 2016, there have been no major catchment net inflows since 1991 (Fig. 2c,d). There were significant precipitation-driven inflows during SON 2010 which led to flood level exceedances at Wagga Wagga and Hay in December 2010. In June and July 1991 there was a series of rain-producing cut-off low pressure systems over inland NSW and the adjacent coast influencing the catchment, interspersed with persistent, precipitation-producing frontal systems embedded in the westerly airflow during July and August. Rain producing inland cut-off low pressure systems over southeast Australia are the main influence on enhancing JJAS rainfall totals^8^.

Decreased JJAS precipitation in continental southeast Australia has been evident for at least the last two decades, as anticipated by climate scientists. The naturally periodic La Niña phenomenon provided spring and summer precipitation during much of 2010 to 2012, which ended the Millennium Drought (1997–2009). The only other recent widespread significant rainfall in southeast continental Australia was in August–September 2016 due to a negative phase of the Indian Ocean Dipole (IOD). A negative IOD phase typically is associated with wetter than normal spring conditions for southeast Australia^7,8^.

**Figure 7:**
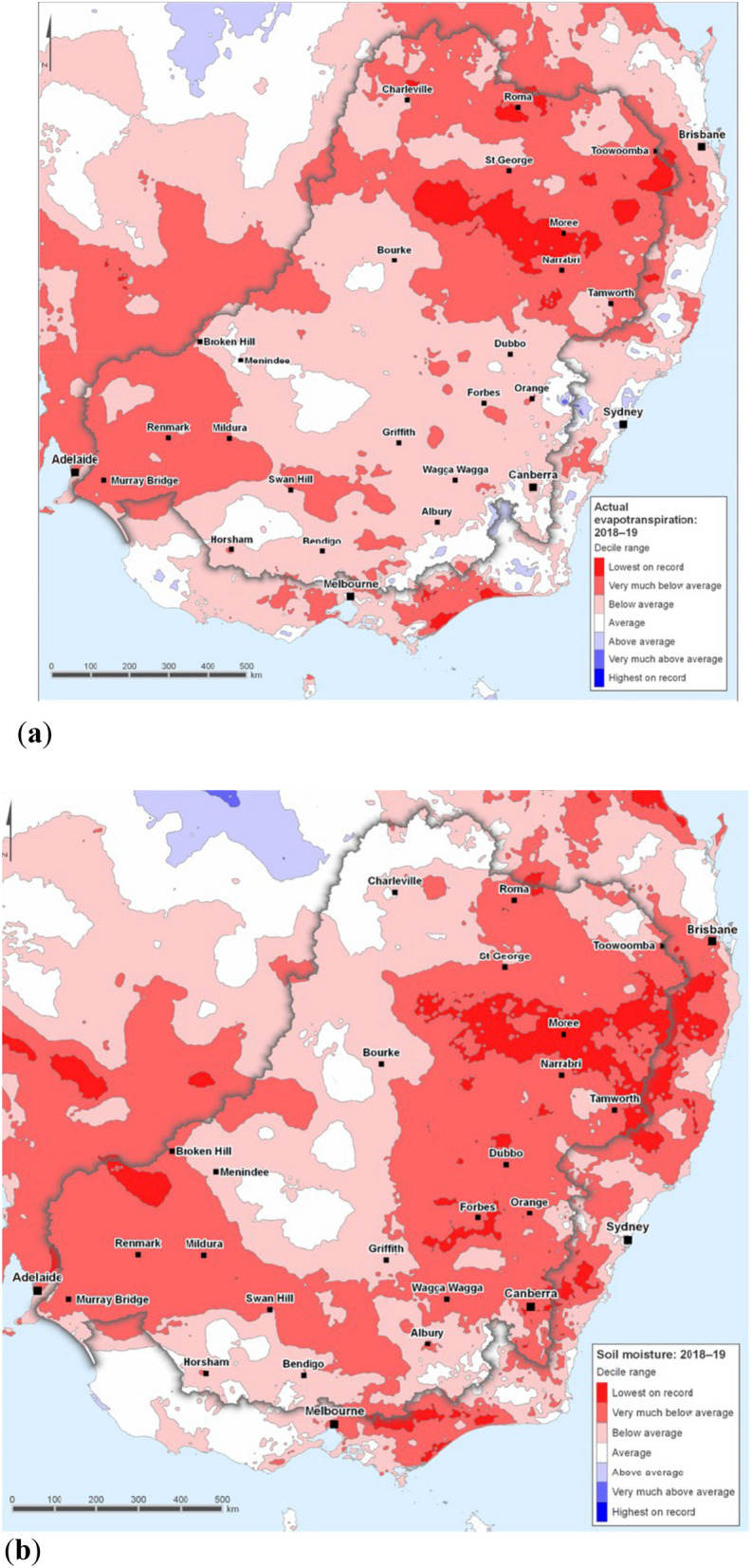
Annual deciles of actual evapotranspiration and soil moisture 2018–2019. Map of southeast Australia showing for the MDB region deciles during the 2018–2019 year for, **(a)** annual area-averaged actual evapotranspiration. Note the below average decile in the southeast corner of the MDB, and **(b)** annual area-averaged soil moisture. Note the very much below average decile in the southeast corner of the MDB. (Reproduced with permission under Creative Commons Attribution Licence 3.0 from the Australian Bureau of Meteorology. Available at: http://www.bom.gov.au/water/nwa/2019/mdb/climateandwater/climateandwater.shtml.

Although the SAM is an atmospheric index with a time scale typically of a few weeks, an annual average SAM reconstruction shows that since the 1970s it is in its most positive state over at least the past 1000 years^27^. Prior to the 1990s soil wetness would have been in phase with the annual cycle of winter/spring peak rainfall, dry summer/early autumn and without a long term trend in SAM. However, because SAM has trended positive since the 1970s, the annual cycle of soil wetness of the MDB has been increasingly disrupted particularly since the Millennium Drought^23^ and there is also a potential long-term impact from groundwater systems^28^. This is supported by the most recent available annual area-averaged actual evapotranspiration and soil moisture deciles in Fig. 7a,b. These figures show the anomalously dry MDB catchment area in the period 2018–2019. In the southeast corner of the MDB, actual evapotranspiration is below average and soil moisture is very much below average.

Two Supplementary Tables showing historical April–May (S1) and JJAS (S2) maximum river heights above flood level at Hay (6.7 m) in IPO phases indicate, as expected, more in negative IPO phases than positive phases and importantly a dissociation with the IPO resulting from none in the most recent negative phase from 1998 and the preceding positive phase after the early 1990s. The implication is that accelerated global warming since the 1990s has overwhelmed the influence of negative IPO on precipitation.

The MDB plan, introduced from 2013^29^ provided, for the first time, regulated allocations to environmental flows for ecosystem sustainability of rivers in southeast Australia such as the Murrumbidgee. However, the plan requires that each year on 1 July a fixed amount of water is locked in for future consumption, split three ways with the highest priority for human consumption and irrigation for permanent crops (e.g., fruit trees and nuts). The remaining allocations are split between non-permanent crops (e.g., cotton, rice) and environmental flow. A major issue is that the forecast net inflows upon which the allocations are based are the minimum inflows experienced in the 120 years up to the end of the twentieth century. However, as shown, even lower inflows have been experienced in the past two decades. It is not surprising that there is a significant decrease in the JJAS variance of the Murrumbidgee River height at Hay (p-value = 0.005) and both the JJAS mean and variance at Wagga Wagga from the periods 1965–1991 to 1992–2018 (mean p-value = 0.0044, variance p-value = 0.095; Table 4) since this period corresponds with the significantly reduced mean April–May catchment precipitation and mean April–May dam net inflows. The fact that there has been no significant change in the mean Murrumbidgee River height since 1991 is an indication that there has been a lack of major April-September rain events. The lack of significant catchment rainfall events from April to September is the reason for the reduction in the mean and variance of river heights at Wagga Wagga. Floods in April–May are rare along the Murrumbidgee River and the six years since 1874 in which April–May floods occurred at Hay prior to 1991 (Table 3), were dominated by precipitation that occurred as a result of mid-latitude interaction with either tropical or subtropical moisture, whereas the last flood that occurred in March 2012, was the result of a rain-producing tropical low pressure trough in the easterly wind regime that extended from northwest Australia to a low pressure centre in southern New South Wales near the Murrumbidgee catchment. Moreover, given the significant decline in April–May, Murrumbidgee catchment rainfall, JJAS run-off into the dams and Murrumbidgee River height at Wagga Wagga since 1991, the implication for water allocations of irrigated agriculture downstream from Wagga Wagga and for flood plain environmental flows required for sustainable wetlands downstream from Hay, will continue to be a problem.

## Conclusions

Both the mean and variability of JJAS river height data at Wagga Wagga and the variability of JJAS river height data at Hay on the Murrumbidgee River in the MDB have decreased significantly since the early 1990s, owing to one only July minor flood level exceedance at Wagga since 1995 and one only September flood level exceedance in 2016. In sharp contrast, the flood level at both Hay and Wagga Wagga was exceeded every few years in JJAS from 1874 to the early 1990s. There has been no new regulatory infrastructure built upstream from Hay since the 1960s. Bootstrapped data box plots for 27-year periods covering JJAS months exhibit no statistically significant decline in the mean catchment precipitation at locations including Burrinjuck Dam, Blowering Dam and Tumut for the period 1992–2018, to match the statistically significant lower mean and variability in river heights at Wagga Wagga or the variability further downstream at Hay. Water extraction for irrigation is unlikely to be a major cause between Wagga Wagga and Hay because the river height had already decreased significantly at Wagga Wagga. However, there is a *highly significant* decline in late autumn (April–May) mean and variance of catchment precipitation between the periods 1965–1991 and 1992–2018. Insufficient late autumn moistening of the dams’ catchment areas reduces run-off during JJAS months even when the mean JJAS rainfall has not declined significantly. A further contributing factor is likely to be the observed mean temperature increase during April-September, thereby increasing evapotranspiration, and reducing run-off into rivers and dams. Total evaporation has increased by up to 2.5 mm per decade since the 1970s. The contributions of decreased April–May precipitation, decreased April–May and JJAS dam net inflows, and increased mean temperatures, which represents the accelerated global warming signal since the 1990s, all reduced catchment area run-off. Future work therefore is planned to address the high priority of searching for attributes, to assist in identifying and understanding the role of the key meteorological drivers, especially those related to the April–May decrease in catchment rainfall.

Regardless of the mandated environmental flows in the last decade and the annually determined sustainable extraction limits on irrigation, water availability from the Murrumbidgee River at Wagga Wagga and Hay over recent decades continues to be affected by changes in catchment precipitation and run-off. In this study the decreased river heights at Wagga Wagga and Hay in the 27-year period, 1992–2018, occurred as a result of a change in seasonality of rainfall and increase in potential evaporation during the current accelerated period of global warming as described above, suggesting the need for a new review of water availability and sustainability in the Murrumbidgee River system and also of other river systems in the southern MDB.

## Methods

The available data sets were Murrumbidgee River heights at Hay in the southern MDB, monthly precipitation, and maximum and minimum screen temperatures at four stations at Blowering Dam, Burrinjuck Dam, Tumut and Cabramurra. The aims of the methodology were to identify trends in the data sets and to use wavelet analysis to identify possible climate drivers of the trends. In addition, ML techniques used to assess the importance of a wide range of climate drivers related to catchment precipitation, are described briefly.

### Trend analysis

The time series data were first plotted with their percentiles to obtain an overview of any trends which might be present in the data. Each data set was grouped into two equal 27-year periods, by taking account of the accelerated global warming signal from the mid-1990s and the archived length of each variable. Hence, the ending period was 1992–2018, and bootstrap resampling was applied with 5000 resamples. Box plots of the mean and variance of the bootstrapped intervals were created which provide a deeper understanding of any trends that might be present in the data. Permutation testing was applied with replacement to test for statistical significance in any apparent changes between two 27-year periods.

### Wavelet analysis

Wavelet analysis^30,31^ was applied to each time series to detect potential climate drivers such as the El-Niño Southern Oscillation. This approach provides both the local wavelet power spectrum (e.g., Fig. 6a, left panel) and the global power spectrum (e.g., Fig. 6a, right panel). The local wavelet power spectrum shows how the influence of climate drivers changes over time, while the global power spectrum provides an overview of which drivers are dominant in the time series. In this study, we used the Morlet wavelet as the mother wavelet.

### Machine learning techniques

Multiple linear regression, support vector regression (with both radial basis function and polynomial kernels), and random forests were used with either forward or backward selection through the space of potential attributes to determine the key attributes of precipitation in the Murrumbidgee catchment. These techniques were employed in a detailed study of the Sydney catchment^32^. By applying tenfold cross-validation on the data set, which covers the years 1965–2018, those attributes that appear in at least 50% of the folds across the eight training methods are considered key attributes of precipitation.

## Supplementary Information

Supplementary Tables.

## Data Availability

The rainfall and temperature station datasets in this study are available online at the Australian Bureau of Meteorology at: http://www.bom.gov.au/climate/change/index.shtml#tabs=Datasets. The digitized weekly river height data at Hay are available at:—https://doi.org/10.5281/zenodo.3779490. Data sets for the SAM, IOD, IPO, ENSO and Global SST time series data were obtained from the NOAA-ESRL Physical Sciences Laboratory, Boulder Colorado from their Web site at: http://www.esrl.noaa.gov/psd/gcos_wgsp/Timeseries/.
