# Supplementary material for: From the 1990s climate change has decreased cool season catchment precipitation reducing river heights in Australia’s southern Murray-Darling Basin
Source: Sci Rep. 2021 Aug 9;11:16136. doi: 10.1038/s41598-021-95531-4 (PMC8352959; doi:10.1038/s41598-021-95531-4)
Supplement: Supplementary file 1 — Supplementary Tables. [file 41598_2021_95531_MOESM1_ESM.pdf]

From the 1990s Climate Change has Decreased Cool Season Catchment Precipitation Reducing River Heights in Australia's Southern Murray-Darling Basin

Authors: **M.S. Speer, L.M. Leslie, S. MacNamara, J. Hartigan**

Year	April	May	April + May	IPO phase	% years of phase
**1888**	6.8		1	negative	9% (2/22 yrs)
**1894**	7.32	8.02	2	negative	"
**1950**	8.56	7.04	2	negative	10% (3/31 yrs)
**1956**	6.89	8.23	2	negative	"
**1974**		6.85	1	negative	"
**1989**	8.14	7.93	2	positive	5% (1/22 yrs)

Table S1. Murrumbidgee river heights at Hay ≥ 6.7 m (minimum flood height) covering years 1874 to 2018 for April-May. Also shown for each year are:- total no. of April-May occurrences; IPO phase; and percentage of years of each IPO phase. Note that most flood height exceedances occur in negative IPO phases and that there have been no flood height exceedances in the negative IPO phase that started from 1998.

Year	Jun	Jul	Aug	Sep	JJAS	IPO phase	% of IPO phase
**1874**		7.92	7.92	7.92	3	negative	50% (11/22 yrs)
**1875**	7.25	7.32			2	negative	"
**1878**				7.77	1	negative	"
**1886**				6.95	1	negative	"
**1887**		7.86			1	negative	"
**1888**				6.71	1	negative	"
**1889**		7.32		6.71	1	negative	"
**1890**		7.32			1	negative	"
**1891**		8.78	7.62		2	negative	"
**1893**		6.86		8.15	2	negative	"
**1894**		6.71	8.38		2	negative	"
**1900**		8.5			1	positive	28% (4/14 yrs)
**1905**			6.74	7.62	2	positive	"
**1906**				7.62	1	positive	"
**1909**				7.09	1	positive	"
**1916**				7.44	1	neutral	42% (5/12 yrs)
**1917**			7.85	8.11	2	neutral	"
**1918**			6.89	7.01	2	neutral	"
**1922**			8.33		1	neutral	"
**1923**				6.71	1	neutral	"
**1925**	8.15	7.77		7.77	3	positive	29% (6/21 yrs)
**1926**			6.81		1	positive	"
**1932**				7.09	1	positive	"
**1934**				7.74	1	positive	"
**1935**			7.39		1	positive	"
**1939**				8.29	1	positive	"
**1949**			7.56		1	negative	29% (9/31 yrs)
**1950**			7.28		1	negative	"
**1951**	8.23	8.56	7.62	6.86	4	negative	"
**1955**	8.31	8.99	8.5	8.15	4	negative	"
**1956**				7.85	1	negative	"
**1960**				6.83	1	negative	"
**1963**			6.93		1	negative	"
**1964**				7.19	1	negative	"
**1974**		7.24	7.32	8.99	3	negative	"
**1983**			7.2		1	positive	23% (5/22 yrs)
**1984**				7.26	1	positive	"
**1989**		6.72	6.8		2	positive	"
**1990**		6.96	7	7.27	2	positive	"
**1991**		7.73			1	positive	"

Table S2. Murrumbidgee river heights at Hay ≥ 6.7 m (minimum flood height) covering years 1874 to 2018 for each June, July, August and September (JJAS) month. Also shown for each year are:- total no. of JJAS occurrences; IPO phase; and percentage of years of each IPO phase. Note that most flood height exceedances occur in negative IPO phases and that there have been no flood height exceedances in the negative IPO phase that started from 1998.
